# IL-7 promotes the formation of DNA double strand breaks and DNA repair in murine pro-B cells

**DOI:** 10.3389/fimmu.2025.1633892

**Published:** 2025-10-06

**Authors:** Alessia Lamolinara, Chiara Di Lisio, Julie A. Hixon, Pasquale Simeone, Antonella De Cola, Maria D. Falco, Thomas J. Meyer, Alessio Ferrone, Domenico Genovesi, Paola Lanuti, Wenqing Li, Vincenzo De Laurenzi, Manuela Iezzi, Francesca B. Aiello, Scott K. Durum

**Affiliations:** ^1^ Department of Neurosciences, Imaging and Clinical Sciences, "G. d'Annunzio" University of Chieti-Pescara, Chieti, Italy; ^2^ Center for Advanced Studies and Technology (CAST), "G. d'Annunzio" University of Chieti-Pescara, Chieti, Italy; ^3^ Department of Medicine and Aging Sciences, "G. d'Annunzio" University of Chieti-Pescara, Chieti, Italy; ^4^ Cytokines and Immunity Section, Cancer Innovation Laboratory (CIL), National Institutes of Health (NIH), National Cancer Institute Frederick, Frederick, MD, United States; ^5^ Department of Oncology, Early Cancer Institute, University of Cambridge, Cambridge, United Kingdom; ^6^ CRUK Children’s Brain Tumour Centre of Excellence, University of Cambridge, Cambridge, United Kingdom; ^7^ Department of Radiation Oncology, "G. d'Annunzio" University of Chieti-Pescara, “S.S. Annunziata” Hospital, Chieti, Italy; ^8^ CCR Collaborative Bioinformatics Resource (CCBR), National Institutes of Health (NIH), National Cancer Institute Frederick, Frederick, MD, United States; ^9^ Department of Innovative Technologies in Medicine and Dentistry, "G. d'Annunzio" University of Chieti-Pescara, Chieti, Italy

**Keywords:** IL-7, pro-B cells, double strand breaks, DNA repair, cell survival

## Abstract

In pro-B cells, VDJ recombination at the immunoglobulin heavy chain locus is impaired. B cell progenitor recombination implies the formation of DNA double strand breaks (DSBs) by the RAG recombinase, which are subsequently repaired by specific mechanisms. We cultured primary murine pro-B cells with IL-7 to evaluate H2AX histone phosphorylation, a well-established marker of DSB formation (γ-H2AX foci) and the expression of proteins involved in DNA repair. Our results indicated that IL-7 upregulated the expression of several molecules involved in homologous recombination, the most accurate DSB repair mechanism. Quantitative analyses of γ-H2AX foci revealed that IL-7 significantly increased DSB formation in a time-dependent manner. Furthermore, γ-H2AX expression was altered in RAG2-deficient pro-B cells and absent in RAG1-deficient pro-B cells treated with IL-7, demonstrating the requirement of both RAG1 and RAG2 recombinase subunits. CD43 expression inversely correlates with the degree of cell differentiation and its level is often evaluated to assess the B lymphoid developmental stage. We observed that IL-7 upregulated CD43 expression and the percentage of large CD43/γ-H2AX double-positive cells, suggesting an effect on less differentiated, immature cells. Notably, we also found that IL-7 increased radiation-induced DSBs, while simultaneously supporting cell survival. This study uncovers novel effects of IL-7 on B cell differentiation, DSB formation, and DNA repair. It is well established that IL-7 promotes the proliferation and survival of acute lymphoblastic leukemia (ALL) cells. Our data suggest that drugs targeting IL-7 could improve ALL therapeutic protocols.

## 1 Introduction

IL-7 is essential in murine and human B cell lymphopoiesis (1–5). B lymphoid cell development requires gene recombination at the immunoglobulin heavy chain (*Ig_H_*) and at κ and λ light chain loci to generate antigen receptor diversity (2, 4). Early and recent studies have shown an impairment of V_H_(D)J_H_ recombination in IL-7 receptor (IL-7R) α chain-deficient pro-B cells (6–8).

IL-7 induces the proliferation of pro-B cells and supports their survival by increasing the expression of anti-apoptotic proteins, including BCL2 and MCL1 (2, 4, 5). Phosphorylation of the transcription factor STAT5 by the tyrosine kinases JAK1 and JAK3, which bind IL-7R α and γ chains, respectively, is indispensable for IL-7-dependent proliferation and survival (2, 9). STAT5 phosphorylation is also required for the expression of the transcription factor EBF1, necessary for B cell lymphopoiesis and for the expression of *Rag1*, encoding the catalytic subunit of the recombinase (3, 10–15). IL-7Rα chain-deficient murine pro-B cells and pro-B cells carrying a point mutation in the α chain at the STAT5 binding site show decreased STAT5 phosphorylation, reduced *Ebf1, Rag1* and *Rag2* gene expression and impaired V_H_(D)J_H_ recombination (16). STAT5-deficient mice exhibit perinatal mortality, atrophic thymus and spleen (2, 9).

The lymphoid specific recombinase, or RAG complex, introduces DNA double strand breaks at the recombination signal sequences, and is formed by two RAG1 catalytic subunits, responsible for the endonuclease activity, and two RAG2 regulatory subunits (17–19). RAG2 is unable to cleave DNA, however, it inhibits RAG1 unbalanced aggregation, promotes the binding of the complex to DNA, and facilitates DNA cleavage and joining steps (17–20). Therefore, both RAG1- and RAG2-deficient mice lack mature T and B cells (21).

Effective recombination, occurring in nuclear “recombination centres”, requires high expression of the recombinase, accessibility of signal sequences that bind the recombinase, and correct three-dimensional structures of DNA loci (17–19). IL-7 induces EBF1, which in turn binds the *Rag1* enhancer and upregulates the expression of the transcription factor PAX5 which increases the transcription of *Rag1* and *Rag2* genes (10–12, 22, 23). In line with these data, obtained in murine experimental models, IL-Rα chain-deficient human pro-B cells show reduced EBF1 and PAX5 protein expression, reduced *Rag1* and *Rag2* gene expression and, interestingly, impaired antisense intergenic transcription, a mechanism that promotes chromatin accessibility (3). The phosphorylation of H2AX histones, surrounding DSBs at serine 139 by ATM, ATR, and DNA-PKc serine-threonine kinases, stabilizes cleaved DNA strands until repair, and is a marker of DSB formation (γ-H2AX foci) (24–26). DNA double strand breaks are subsequently repaired by specific mechanisms (17–19). Two major pathways are involved in DSB repair: non-homologous end joining (NHEJ), and homologous recombination (HR) (25). NHEJ operates throughout the cell cycle independent of homology, and promotes direct ligation of DSBs, thus, it can introduce insertions, deletions, substitutions and translocations at break sites (25). HR, functioning in the late S and G2 phases, uses an identical undamaged sister chromatid or a chromosome homologue as a template, ensuring accurate repair (25). The efficiency of DNA repair depends on the activity of Poly ADP-ribose (PAR) polymerase 1 (PARP1), a key member of a family of enzymes that functions at DNA damaged sites. PARP1 transfers PAR chains to HR and NHEJ effector proteins and is frequently overexpressed in cancer (27, 28).

How IL-7 promotes V_H_(D)J_H_ recombination is not entirely clear, and whether IL-7 would influence DNA repair has not been studied. Therefore, we aimed to investigate the effects of IL-7 on DSB formation and DNA repair. Notably, IL-7 increases the proliferation and survival of IL-7R positive acute lymphoblastic leukemia (ALL) cells, as it will be discussed (2). A better understanding of these mechanisms may contribute to the development of novel targeted therapies for ALL.

## 2 Methods

### 2.1 Mice

C57BL/6 and RAG1-deficient mice were obtained from the Animal Facility of the National Cancer Institute (MD, USA). Animal care was provided in accordance with the procedures outlined in the “Guide for Care and Use of Laboratory Animals” (NIH, Bethesda, USA). BALB/c mice were obtained from the Animal Facility of the Center for Advanced Studies and Technology (“G. d’Annunzio” University of Chieti-Pescara). RAG2-deficient mice were obtained from The Jackson Laboratory (ME, USA). All procedures were approved by the Ethic Committee of the Italian Ministry of Health (authorization n° 338/2018-PR).

### 2.2 Preparation of cells

Cells were cultured in RPMI 1640 supplemented with 10% fetal bovine serum (FBS) (Hyclone, Logan, UT), 2mM L-glutamine, 100 U/ml penicillin, 100 mg/ml streptomycin and 50 mM β-mercaptoethanol (Invitrogen, CA, USA). For microarray analysis, bone marrow cells from C57BL/6 mice were negatively selected by cell sorting using the following monoclonal antibodies (moAbs): Fitc-anti-GR-1 and anti-TER-119 moAbs, PE-anti-MAC-1 moAb, Fitc-anti-IgM and anti-CD3 moAbs (BD Biosciences, CA, USA). Cells separated using a MoFlo high speed cell sorter (Dako Cytomation, Fort Collins, Co) yielded 90% B220+ CD19+ IgM- cells (29). For flow cytometry and confocal microscopy, bone marrow cells from C57BL/6, BALB/c, RAG1- and RAG2-deficient mice were depleted from IgM expressing B cells by negative selection using anti-rat microbeads recognizing rat-anti-IgM moAb (BD Biosciences) and LS MACS columns (Miltenyi Biotech, Germany). IgM depleted bone marrow cells were stained with rat anti-B220 moAb (BD Biosciences) and submitted to positive selection using LS MACS columns (Miltenyi Biotech). This procedure yielded 80-86% B220+ CD19+ IgM- cells. Viability, evaluated by trypan blue staining was higher than 90%. After 96 and 192 hours of IL-7 treatment, the percentages of dead cells were 9.0±1.4 and 32.40±1.80, respectively (mean ± SEM). Murine IL-7 was always used at the concentration of 50 ng/ml (Peprotech Inc, Rocky Hill, NJ) as indicated in Figure Legends.

### 2.3 RNA extraction and microarray analysis

Total RNA was extracted from purified pro-B cells as previously described (29) and residual DNA was digested using the RNA easy kit (Qiagen, CA, USA). Quality of triplicate RNA samples was evaluated using the RNA Integrity Number (RIN) on the Agilent Bioanalyzer. Microarray analysis was performed using the low-input Affymetrix Gene Chip Mouse Genome 430 2.0. The low-input microarray was performed at the Laboratory of Molecular Technology (Leidos Biomedical Research Inc, MD, USA). The Gene Expression module workflow was executed for analysis of the raw data that passed all initial quality checks, including principal component analysis (PCA). Differential gene expression was assessed using Analysis of Variance (ANOVA) test to compare between the respective sample groups. The Affymetrix CEL files, representing triplicates for each of the experimental groups, were uploaded onto Partek Genomics Suite v6.6. To compare samples treated with or without IL-7, the default statistical cutoffs of adjusted *p* value (False Discovery Rate, FDR) < 0.05 and fold-change of > 2 or < -2 were applied to generate a list of significantly differentially expressed genes. Zero genes failed to pass the nominal *p* value < 0.05.

### 2.4 Quantitative real-time PCR

mRNA from pro-B cells (1mg) was reverse transcribed using TaqMan Reverse Transcription Reagents (Applied Biosystem, MA, USA). Real-time PCR was performed using the SsoAdvanced Universal SYBR Green Supermix. The amplification program was: 30 sec at 95°C, 15 sec at 95°C and 30 sec at 55°C. The reaction was followed by a melting curve protocol according to the specification of the CFX96 Real Time PCR System (Bio-Rad, CA, USA). Gene expression was normalized to β-actin and relative quantification was calculated according to 2-^ΔΔCT^ comparative method. Primers are reported in **Supplementary Table 1**.

### 2.5 Western blot analysis

Western blot analysis was performed as previously described (29). The following antibodies were used: rabbit anti-MYC, anti-β-ACTIN and anti-RAD51 monoclonal antibodies (MoAbs) (Cell Signaling, MA, USA), mouse anti-BARD1 and anti-BRIP1 MoAbs (Santa Cruz, CA, USA), mouse anti-PLK1 MoAb (Millipore, Germany), and correspondent polyclonal HRP-conjugated secondary antibodies (Cell Signaling).

### 2.6 Immunofluorescence analysis

Pro-B cells were cultured with or without IL-7 for 24, 48 and 72 hours. Due to decreased viability, cells were not maintained beyond 72 hours in the absence of IL-7. In the presence of IL-7, cell cultures were extended to 144 and 192 hours. Slides were prepared with approximately 3x10^5^ cells per EZ cyto-funnel chamber (Fisher Scientific, NH, USA). Slides were fixed in 4% paraformaldehyde for 5 minutes, washed three times with PBS for 5 minutes and then permeabilized with PBS/0.1% Triton X for 5 minutes. Then, slides were washed three times with PBS for 5 minutes, incubated with PBS/1% BSA for 30 minutes and with murine anti-γH2AX MoAb (Ser 139) (Millipore) at 4°C overnight. Slides were washed three times with PBS for 5 minutes and incubated with Alexa488-conjugated polyclonal goat anti-mouse antibody (Invitrogen) for 1 hour, at room temperature, followed by nuclear staining with DAPI (Sigma) for 15 minutes.

Images were acquired using a Zeiss LSM800 confocal microscope. Quantification of γ-H2AX staining was performed on digital images using Adobe Photoshop 13.0 software. For each field, nuclei were selected and analyzed for positive green-fluorescent pixels. Cells showing a nuclear fluorescence intensity > 100 pixels were considered positive.

### 2.7 Irradiation

Pro-B cells underwent direct radiation exposure by a 6-MV linear accelerator (Elekta Synergy^®^, Crawley, UK). A planning computed tomography (CT) scan was performed using Somatom Emotion ^®^ CT scanner (four slices, Siemens, Germany). Cells, seeded in 24 well plates, were delineated as targets by a radiation oncologist and received doses from 0.25 to 1.5 Gy/plate. The 24 well plates were placed at the center of a self-made phantom. A 5-cm RW3 solid phantom (ρ = 1.045 g/cm^3^) provided the backscatter radiation. From CT image sets, a personalized treatment plan in isocenter technique was developed (30x30 cm^2^ field size, 6-MV photon energy, and a source-to-surface distance of 97 cm). Radio-opaque markers were used to achieve correct phantom repositioning in each single session.

### 2.8 Flow cytometry

Pro-B cells were incubated with PBS/0.5% BSA for 15 minutes at 4°C, fixed and permeabilized using Cytofix/Cytoperm solution (BD Biosciences). Cells were incubated with Alexa488-conjugated rabbit anti-γH2AX MoAb (Cell Signaling) for 30 minutes at room temperature, or in alternative with an unconjugated rabbit anti-γH2AX MoAb (Cell Signaling) for 30 minutes at room temperature, followed by an incubation with a polyclonal goat anti-rabbit Alexa488-conjugated antibody (Invitrogen). For two colors staining, after washing, cells were incubated with APC-conjugated anti-CD43 MoAb recognizing the 115 kDa isoform (BD, Franklin Lakes, NJ. USA) for 30 minutes. At least 20.000 events/sample were acquired by flow cytometry (FACSCanto II, BD Biosciences) at “medium” flow rate mode. Data were analyzed using FlowJo v10.10 software (BD Biosciences). Forward and Side Scatter values were used to evaluate cell size and internal complexity, respectively. LIVE/DEAD™ Fixable Aqua Dead Cell Stain Kit (Invitrogen) was used to evaluate live/dead cells. Normalized mean fluorescence intensity (MFI) was calculated and expressed as ratio between IL-7-treated and untreated cells.

### 2.9 Statistical analysis

Data are expressed as mean ± SD or SEM as indicated in Figure Legends. A *P* value < 0.05 was considered statistically significant. Statistical differences were calculated with unpaired Student *t* test and linear regression (Pearson’ correlation test). ANOVA test was used for group comparison.

## 3 Results

### 3.1 IL-7 upregulates the expression of genes involved in DNA repair

**Figure 1:**
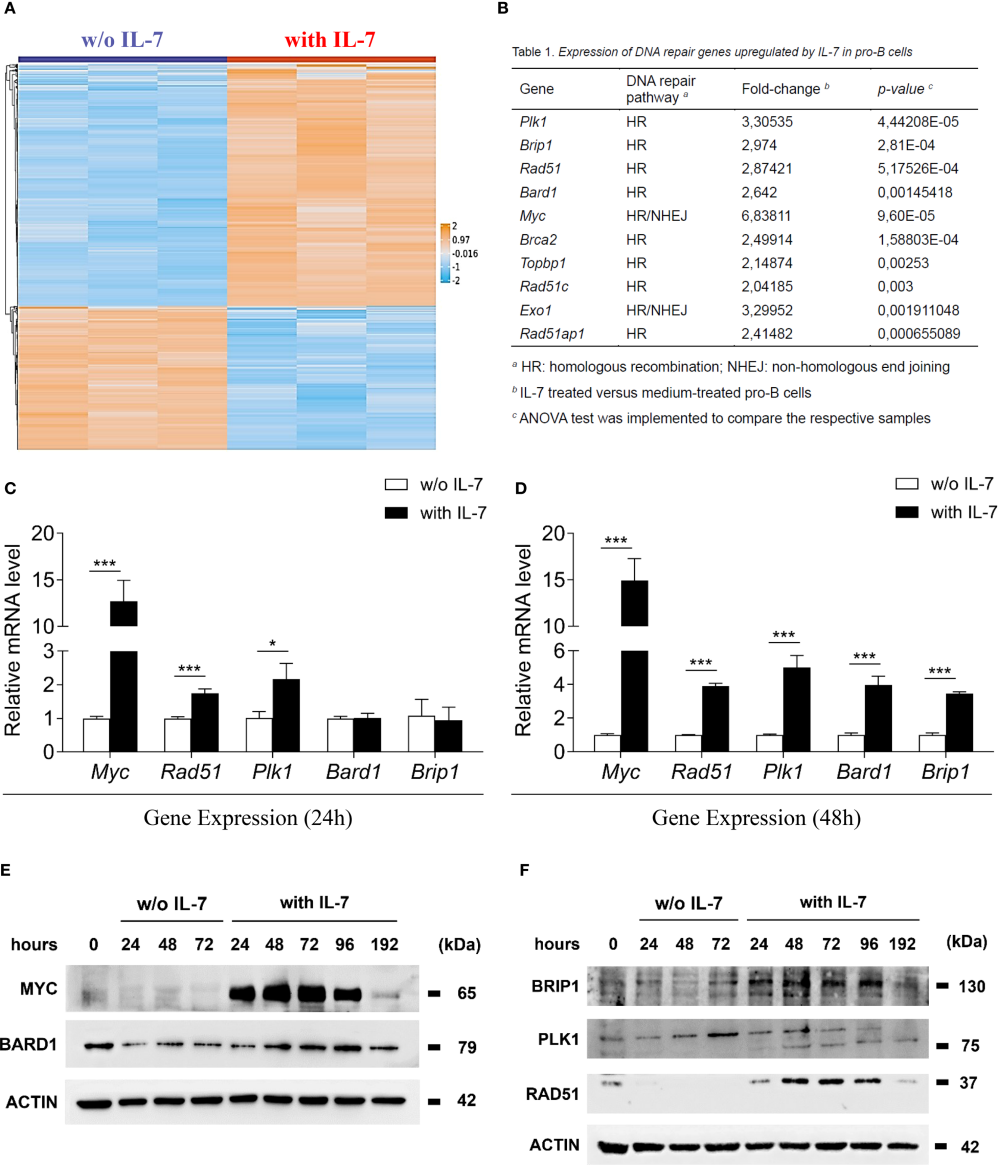
IL-7 upregulated the expression of molecules involved in DNA repair. **(A)** Heat map representation of microarray analysis of pro-B cells treated with IL-7 (50 ng/ml) or without IL-7 for 67 hours, including 1159 differentially regulated genes, showing a > 2-fold increase (orange) or a < 2-fold decrease (blue). Results from 3 experiments. Gene titles, fold changes and *P* values are reported in **Supplementary Table 2**. ANOVA test was used for group comparison. **(B)** DNA repair genes upregulated by IL-7. **(C)** Relative abundance of *Myc, Rad51, Plk1, Bard1 and Brip1* mRNAs evaluated by RT-qPCR after 24 hours treatment without IL-7 (white bars) or with IL-7 (black bars). Results from 3 experiments are shown as mean ± SD. Unpaired *t* test ***P < 0.001, and *P < 0.05. **(D)** Relative abundance of *Myc, Rad51, Plk1, Bard1 and Brip1* mRNAs at 48 hours. Results from 3 experiments are shown as mean ± SD. Unpaired *t* test ***P < 0.001. **(E)** Immunoblot analysis using monoclonal antibodies (MoAbs) recognizing MYC, BARD1 and β-ACTIN at the indicated times. **(F)** Immunoblot analysis using MoAbs recognizing BRIP1 (130 kDa upper band), PLK1 (75 kDa lower band), RAD51 and β-ACTIN at the indicated times. The analysis was not performed on cells cultured without IL-7 beyond 72 hours due to reduced cell viability. Data represent 1 of 3 experiments.

In a previous study, we identified 1202 IL-7-regulated transcripts in murine pro-B cells and showed the hierarchical clustering of 187 genes with greater than four-fold increase or decrease of expression (29). After a gene annotation update, we hierarchically clustered 1159 genes - 725 upregulated and 434 downregulated (listed in **Supplementary Table 2**) - in a heat map showing differentially expressed genes with an increase or a decrease greater than two-fold (**Figure 1A**). IL-7 significantly upregulated the expression of genes involved in HR (*Plk1*, *Brip1*, *Rad51*, *Bard1*, *Brca2*, *Topbp1*, *Rad51c* and *Rad51ap1)*, and genes involved in both HR and NHEJ (*Myc* and *Exo1)* (**Figure 1B**). We validated the expression of 5 genes by quantitative Real-Time PCR (**Figures 1C, D**) and the expression of the corresponding proteins by western blot analysis (**Figures 1E, F**). Following treatment with IL-7, the mRNA levels of *Myc* and *Rad51* was markedly increased at 24 and 48 hours (*** *P* < 0.001; **Figures 1C, D**, respectively). *Plk1* mRNA was slightly increased at 24 hours (* *P* < 0.05; **Figure 1C**) and greatly increased at 48 hours (*** *P* < 0.001; **Figure 1D**), while the upregulation of *Bard1* and *Brip1* mRNA was detected at 48 hours (*** *P* < 0.001; **Figure 1D**). Time courses of mRNA and protein expression were comparable. Western blot analyses and densitometry relative to protein expression are shown in **Figures 1E, F** and in **Supplementary Figure 1**, respectively. MYC and RAD51 protein level increased up to 48 hours, progressively declining at 72, 96 and 192 hours; BARD1 and BRIP1 protein upregulation was evident at 48 hours, their expression increased up to 96 hours, declining at 192 hours (**Figures 1E, F**). PLK1 protein expression was barely detectable at 24 hours, increased at 48 hours and persisted until 192 hours (**Figure 1F**, 75 kDa band). Freshly isolated pro-B cells (0 hours) had low basal protein levels, which, without IL-7 treatment, progressively decreased (**Figures 1E, F**). The analysis was not performed on cells cultured without IL-7 beyond 72 hours due to reduced cell viability. After 96 and 192 hours of IL-7 treatment, the percentages of dead cells were 9.50±1.04 and 32.40±1.80, respectively (mean ± SEM).

### 3.2 IL-7 increases the formation of γ-H2AX foci

**Figure 2:**
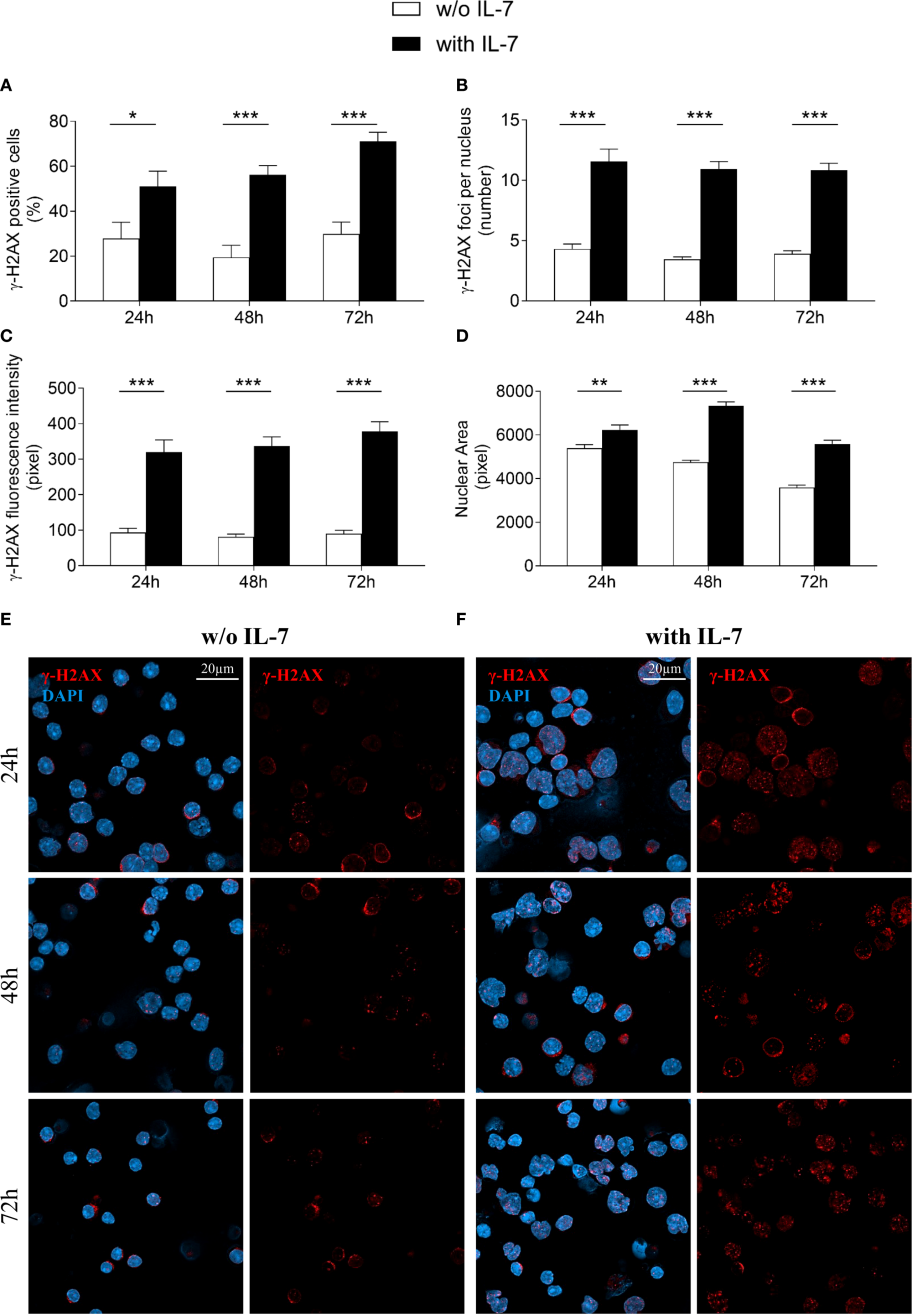
IL-7 increased the formation of double strand breaks. Pro-B cells, cultured with IL-7 (50 ng/ml) or without IL-7, were assessed by confocal microscopy for H2AX histone phosphorylation (γ-H2AX foci) at the indicated times. **(A)** Percentage of γ-H2AX positive cells. **(B)** Mean number of γ-H2AX foci per nucleus. **(C)** γ-H2AX fluorescence intensity. **(D)** Mean nuclear area. **(E)** Representative images of γ-H2AX immunofluorescence staining of untreated pro-B cells. **(F)** Representative images of γ-H2AX immunofluorescence staining of IL-7 treated pro-B cells. γ-H2AX foci in red, nuclei in blue. γ-H2AX staining alone on the right, merge on the left. Results are mean of 3 experiments ± SEM. The number of cells analyzed in the three independent experiments is: with IL-7 n= 224, 348 and 269 at 24h, 48h and 72h, respectively; without IL-7 n= 202, 320 and 173 at 24h, 48h and 72h, respectively. Unpaired *t* test ***P < 0.0001, **P < 0 .005 and *P < 0.05. Scale bar: 20 µm.

IL-7 is required for T cell receptor γ locus accessibility to the V(D)J recombinase in thymocytes and for V(D)J rearrangement at the *Ig_H_* chain locus in B cell progenitors (4, 6, 8, 30). We investigated the effect of IL-7 on the formation of γ-H2AX foci in pro-B cells using confocal microscopy, to quantify even modest DNA damage with high sensitivity (26). IL-7 treatment increased the percentage of γ-H2AX positive cells (* *P* < 0.05, *** P < 0.0001; **Figure 2A**), the number of γ-H2AX foci per nucleus (*** *P* < 0.0001; **Figure 2B**), and the γ-H2AX fluorescence intensity (*** *P* < 0.0001; **Figure 2C**) at 24, 48 and 72 hours. IL-7 also increased the size of the nuclear area (** *P* < 0.005, *** P < 0.0001; **Figure 2D**).

Representative images show untreated (**Figure 2E**) and IL-7-treated (**Figure 2F**) pro-B cells stained for γ-H2AX at the indicated time points.

Due to decreased viability, cells were not maintained beyond 72 hours in the absence of IL-7.

The extent of γ-H2AX foci formation in the presence of IL-7 at 96 and 144 hours was comparable (**Supplementary Table 3**).

**Figure 3:**
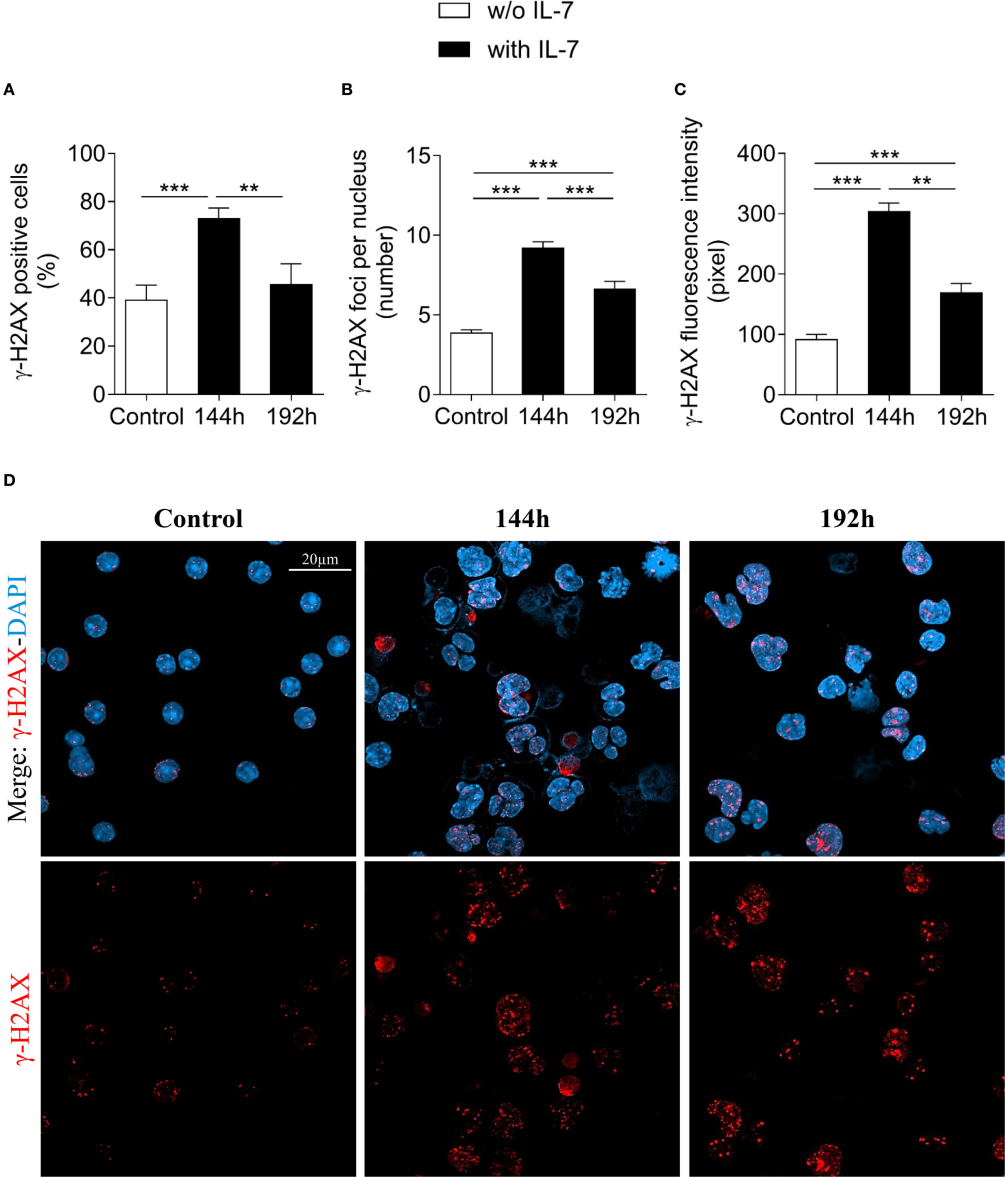
Evaluation of γ-H2AX foci in pro-B cells after extended IL-7 treatment. γ-H2AX foci were evaluated in pro-B cells cultured with IL-7 (50 ng/ml) for 144 and 192 hours. **(A)** Percentage of γ-H2AX positive cells. **(B)** Mean number of γ-H2AX foci per nucleus. **(C)** γ-H2AX fluorescence intensity. **(D)** Representative images of γ-H2AX immunofluorescence staining of untreated (control = freshly isolated) and IL-7 treated pro-B cells at 144 and 192 hours. γ-H2AX foci in red, nuclei in blue. γ-H2AX staining alone in the lower panel, merge in the upper panel. Results are mean of 3 experiments ± SEM. The number of cells analyzed in the three independent experiments is: Control n= 140, 144h n= 477 and 192h n= 402. Unpaired *t* test ***P< 0.001, **P < 0.01. The analysis was not performed on cells cultured without IL-7 beyond 72 hours due to reduced cell viability. Scale bar: 20 µm.

**Figure 3** shows the results obtained after 144 and 192 hours of IL-7 treatment. The percentage of γ-H2AX positive cells, the number of γ-H2AX foci per nucleus and the γ-H2AX fluorescence intensity at 144 hours were still markedly higher than in freshly isolated control cells (*** *P* < 0.001; **Figures 3A–C**, respectively). At 192 hours the percentage of positive cells was similar to control cells (**Figure 3A**), while the number of foci and the γ-H2AX fluorescence intensity were still higher (*** *P* < 0.001; **Figures 3B, C**). Notably, at 192 hours the percentage of γ-H2AX positive cells, the number of foci per nucleus and the γ-H2AX fluorescence intensity were significantly decreased when compared to 144 hours (** *P* < 0.01; **Figure 3A**, *** *P* < 0.001; **Figure 3B**, ** *P* < 0.01; **Figure 3C**, respectively). Representative images show control and IL-7-treated pro-B cells stained for γ-H2AX at 144 and 192 hours (**Figure 3D**).

We also observed a positive correlation between the size of the nuclear area and γ-H2AX fluorescence intensity at all time points (**Supplementary Figure 2**). The increase of nuclear area was accompanied by a time-dependent increase of the cell size, as measured by flow cytometry (**Supplementary Figure 3**). We observed two different cell populations, that, based on the cell size, were gated as large and small pro-B cells. In cells treated with IL-7 the percentage of large cells increased from 15.77 ± 2.62% to 22.45 ± 5.56% at 24 hours (* *P* < 0.05; **Supplementary Figure 3** upper panel), from 9.23 ± 0.92% to 32.65 ± 2.48% at 48 hours (*** *P* < 0.0001; **Supplementary Figure 3** middle panel) and from 10.01 ± 1.05% to 35.72 ± 2.07% at 72 hours (*** *P* < 0.0001; **Supplementary Figure 3** lower panel). Conversely, the percentage of small cells decreased from 82.65 ± 2.91% to 75.67 ± 5.66% at 24 hours (* *P* < 0.05; **Supplementary Figure 3** upper panel), from 90.07 ± 0.96% to 65.48 ± 2.57% at 48 hours (*** *P* < 0.0001; **Supplementary Figure 3** middle panel) and from 88.38 ± 1.82% to 62.62 ± 2.19% at 72 hours (*** *P* < 0.0001; **Supplementary Figure 3** lower panel).

### 3.3 IL-7 modifies the expression of CD43: relationship with γ-H2AX expression

**Figure 4:**
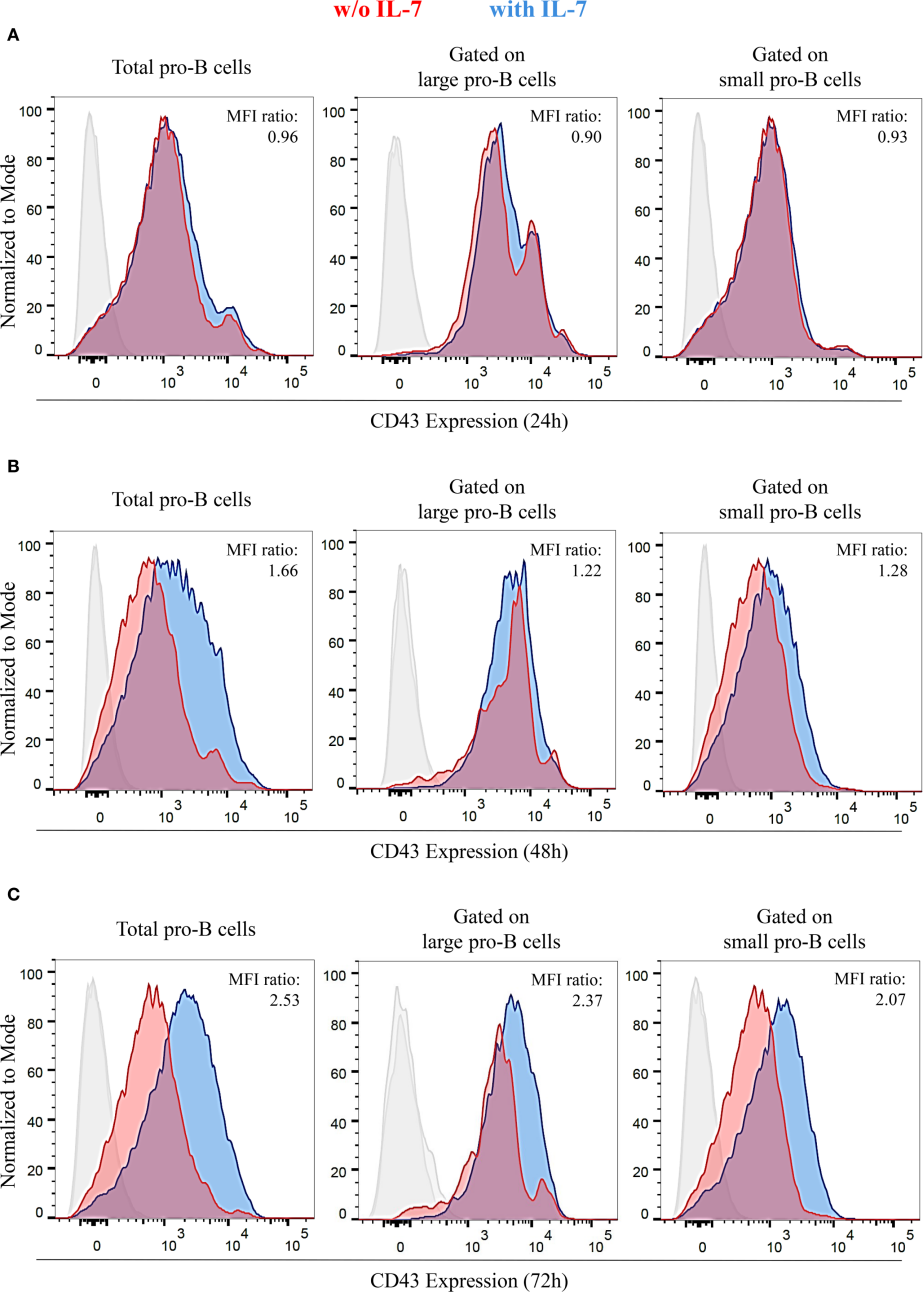
IL-7 upregulated CD43 expression in pro-B cells. CD43 expression in pro-B cells treated without or with IL-7 (50 ng/ml) was analyzed by flow cytometry. Results represent 1 of 3 experiments. **(A)** Cells cultured without IL-7 (red) or with IL-7 (blue) for 24 hours. **(B)** Cells cultured with or without IL-7 for 48 hours. **(C)** Cells cultured with or without IL-7 for 72 hours. Normalized MFI ratios at 24 hours are ≤ 1 in total, large and small cells; normalized MFI ratios at 48 hours are 1.66 (***P < 0.0001), 1.22 (**P < 0.005), and 1.28 (**P < 0.01) in total, large and small cells, respectively; normalized MFI ratios at 72 hours are 2.53 (***P < 0.001), 2.37 (***P < 0.001), and 2.07, (***P < 0.001) in total, large and small cells, respectively. Unpaired *t* test.

Differentiating pro-B cells progressively downregulate CD43 surface expression, so that small pre-B cells, immature and mature B cells are CD43 negative (31, 32). The evidence of pro-B cells exhibiting different cell sizes after IL-7 treatment (**Supplementary Figure 3**) led us to assess the effect of IL-7 on CD43 expression by flow cytometry. We measured CD43 MFI of total, large and small pro-B cells at 24, 48 and 72 hours and calculated the ratio between IL-7-treated and untreated cells (**Figure 4**). Results from three independent experiments are shown in **Supplementary Table 4**. After IL-7 treatment for 24 hours there was no difference in CD43 expression, as confirmed by a normalized MFI ratio ≤ 1 (**Figure 4A**). At 48 and 72 hours a significant and progressive increase of CD43 expression was evident in total, large and small cells (**Figures 4B, C**). Thus, a time-dependent decrease in CD43 expression was not observed. The gates used to separate live/dead cells and large/small cells at 72 hours are shown in **Supplementary Figure 4**.

**Figure 5:**
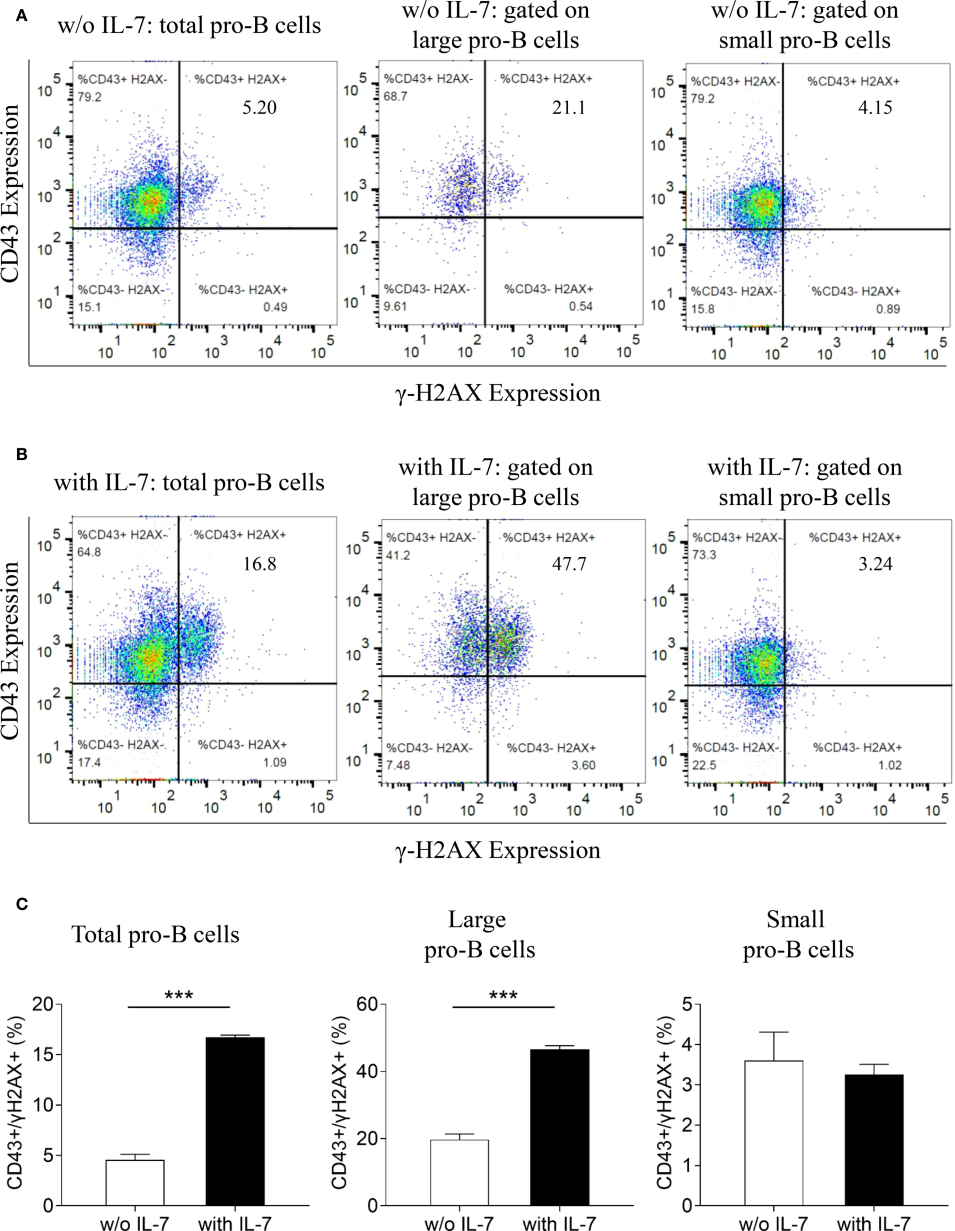
Relationship between γ-H2AX and CD43 antigen expression in pro-B cells treated with IL-7. γ-H2AX and CD43 expression in pro-B cells treated without or with IL-7 (50 ng/ml) was analyzed by flow cytometry. Results represent 1 of 3 experiments. **(A)** CD43/γ-H2AX double staining of pro-B cells cultured for 48 hours without IL-7. **(B)** CD43/γ-H2AX double staining of pro-B cells cultured with IL-7 for 48 hours. **(C)** Bar graphs showing the percentage of CD43/γ-H2AX double positive cells in total, large and small pro-B cells. IL-7 treatment significantly increased the percentage of total CD43/γ-H2AX double positive cells from 4.55 ± 0.57% to 16.73 ± 0.21% (mean ± SD, ***P < 0.001) and the percentage of large double positive cells from 19.73 ± 1.65% to 46.57 ± 1.10% (mean ± SD, ***P < 0.001). Unpaired *t* test.

We then analyzed the expression of γ-H2AX and CD43 in untreated (**Figure 5A**), or IL-7-treated cells (**Figure 5B**) by flow cytometry. As shown by the bar graphs in **Figure 5C**, IL-7 treatment for 48 hours increased the percentage of total and large CD43/γ-H2AX double-positive cells (*** *P* < 0.0001). No increase was detected in small double-positive cells. In addition, a significant increase of γ-H2AX MFI was detected in total and large CD43/γ-H2AX double-positive cells but not in small CD43/γ-H2AX pro-B cells treated with IL-7 (not shown).

### 3.4 Expression of γ-H2AX and formation of foci in RAG1- and RAG2-deficient pro-B cells

**Figure 6:**
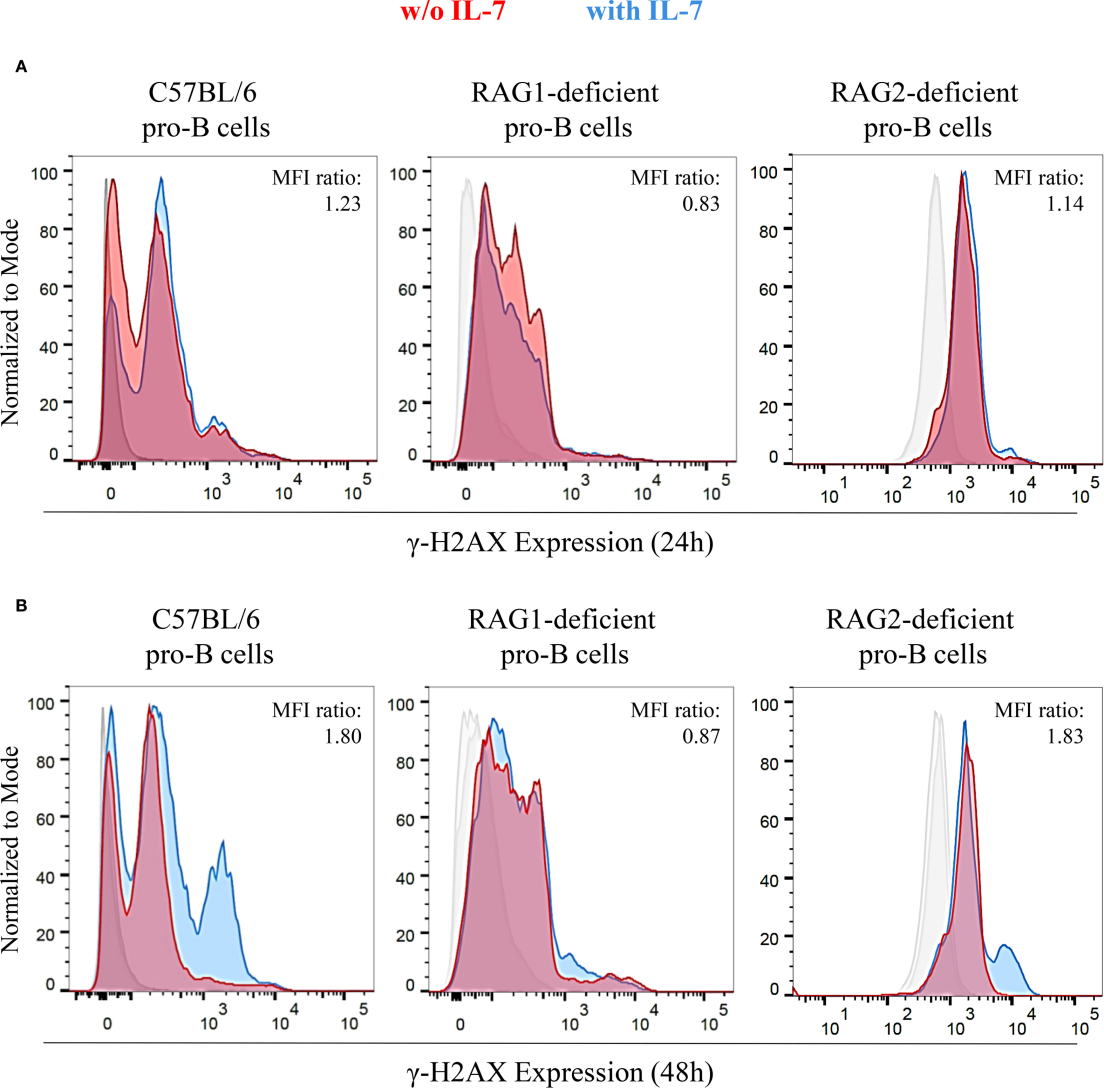
Expression of γ-H2AX in untreated and IL-7 treated pro-B cells from C57BL/6, RAG1- and RAG2-deficient mice. γ-H2AX expression in IL-7 treated (50 ng/ml) or untreated pro-B cells from C57BL/6, RAG1- and RAG2-deficient mice was evaluated by flow cytometry. Results represent 1 of 3 experiments. **(A)** Pro-B cells cultured without IL-7 (red) or with IL-7 (blue) for 24 hours. **(B)** Pro-B cells cultured without or with IL-7 for 48 hours. At 24 hours, C57BL/6 pro-B cells cultured with IL-7 showed a higher γ-H2AX MFI (23.32 ± 1.56) compared to the untreated ones (19.02 ± 0.51). In RAG1-deficient pro-B cells, IL-7 did not modify γ-H2AX MFI (MFIs were 7.07 ± 0.28 without IL-7 vs. 5.86 ± 0.29 with IL-7). RAG2-deficient pro-B cells with IL-7 showed a higher γ-H2AX MFI (3.39 ± 0.26) compared to the untreated ones (2.97 ± 0.32). These differences yielded MFI ratios in pro-B cells from C57BL/6, RAG1- and RAG2-deficient mice of 1.23 (*P* = ns), 0.83 (*P* = ns), 1.14 (*P* = ns), respectively. At 48 hours, the γ-H2AX MFI was significantly higher in IL-7–stimulated C57BL/6 pro-B cells (21.71 ± 0.27) compared to unstimulated cells (12.05 ± 0.02). Similar results were observed in RAG2-deficient pro-B cells (MFI were 2.68 ± 0.11 without IL-7 vs 4.91 ± 0.20 with IL-7). In RAG1-deficient pro-B cells there was no difference due to IL-7 treatment in the MFI (MFIs were 6.30 ± 0.33 without IL-7 vs. 5.48 ± 0.32 with IL-7). The corresponding MFI ratios were 1.80 (***P = 0.0004), 1.83 (**P = 0.005) and 0.87 (*P* = ns), respectively. Unpaired *t* test.

The RAG complex generates the DSBs necessary for recombinase activity (17–19). Consequently, both RAG1- and RAG2-deficient mice show complete lack of mature B and T cells (21). In IL-7R-deficient mice, V_H_(D)J_H_ joining is impaired, and B cell lymphopoiesis is inhibited at an early stage (6, 8). IL-7 increased the cell size of both RAG1- and RAG2-deficient pro-B cells, that remain viable for up to 48 hours. Then, their cell number gradually decreased due to apoptosis, resulting from the lack of productive rearrangements (data not shown). This is consistent with previous results concerning RAG2-deficient mice (33). We investigated whether IL-7 affected γ-H2AX expression in pro-B cells isolated from wild type C57BL/6 mice, RAG1- and RAG2-deficient mice (C57BL/6 genetic background) by flow cytometry (**Figure 6**). We observed a time-dependent increase of the γ-H2AX MFI ratio in IL-7-treated C57BL/6 cells (**Figures 6A, B**), and in IL-7-treated BALB/c pro-B cells (not shown). No increase was observed in RAG1-deficient pro-B cells (**Figures 6A, B**). Unexpectedly, we observed a time-dependent increase of γ-H2AX MFI ratio in IL-7-treated RAG2-deficient pro-B cells (**Figures 6A, B**).

**Figure 7:**
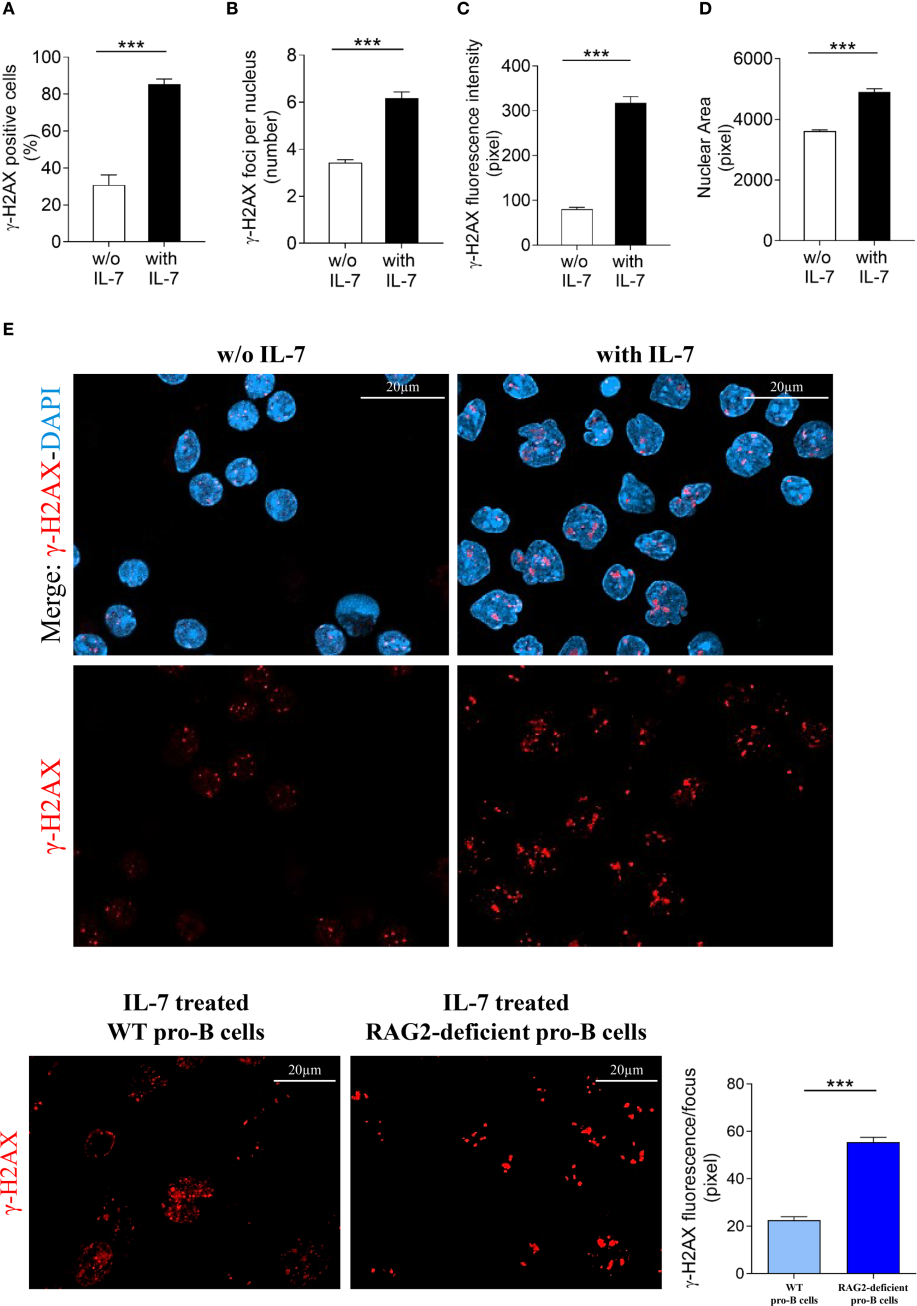
Evaluation of γ-H2AX foci in RAG2-deficient pro-B cells. Pro-B cells from RAG2-deficient mice were cultured without or with IL-7 (50 ng/ml) for 24 hours and analyzed by confocal microscopy. **(A)** Percentage of γ-H2AX positive cells. **(B)** Mean number of γ-H2AX foci per nucleus. **(C)** γ-H2AX fluorescence intensity. **(D)** Mean nuclear area. **(E)** Representative images of γ-H2AX immunofluorescence staining of pro-B cells untreated (left panel) or treated with IL-7 (right panel). γ-H2AX foci in red, nuclei in blue. Lower panel: γ-H2AX staining alone, upper panel: merge. The number of cells analyzed in the three independent experiments is: with IL-7 n= 348, without IL-7 n= 251. **(F)** Left panel: representative images of γ-H2AX foci in IL-7 treated pro-B cells from wild type (WT) and RAG2-deficient mice stained in red. Right panel: quantification of γ-H2AX fluorescence intensity per focus. Results represent mean of three experiments ± SEM. The number of cells analyzed in the three independent experiments is: WT n= 224, RAG2-deficient n= 348. Unpaired *t* test ***P < 0.0001. Scale bar: 20 µm.

Then, to obtain morphologically detailed results, we evaluated γ-H2AX foci formation in pro-B cells from RAG2-deficient mice by confocal microscopy and compared these results with those obtained in cells from wild type mice (**Figure 2**). Unexpectedly, IL-7 treated RAG2-deficient pro-B cells showed a higher percentage of γ-H2AX positive cells than untreated cells (*** *P* < 0.0001; **Figure 7A**) and this increase was more prominent than in wild type cells (85% versus 51%, *** *P* < 0.0001). The number of foci per nucleus also increased (*** *P* < 0.0001; **Figure 7B**), but to a level significantly lower than that observed in wild type cells (6.1 versus 11.5 per nucleus, *** *P* < 0.0001). IL-7 significantly increased γ-H2AX fluorescence intensity (*** *P* < 0.0001; **Figure 7C**) and the nuclear area (*** *P* < 0.0001; **Figure 7D**) of both RAG2-deficient and wild type pro-B cells to a similar extent. Representative images of γ-H2AX foci in untreated and IL-7-treated RAG2-deficient pro-B cells at 24 hours are shown in **Figure 7E**. When we considered the amount of γ-H2AX fluorescence per focus, we observed that IL-7 induced a greater increase of this parameter in cells from RAG2-deficient mice than in cells from wild type mice (55.5 versus 22.4 pixels, *** *P* < 0.0001; **Figure 7F**).

### 3.5 IL-7 increases the formation of γ-H2AX foci and protects cell survival in irradiated pro-B cells

**Figure 8:**
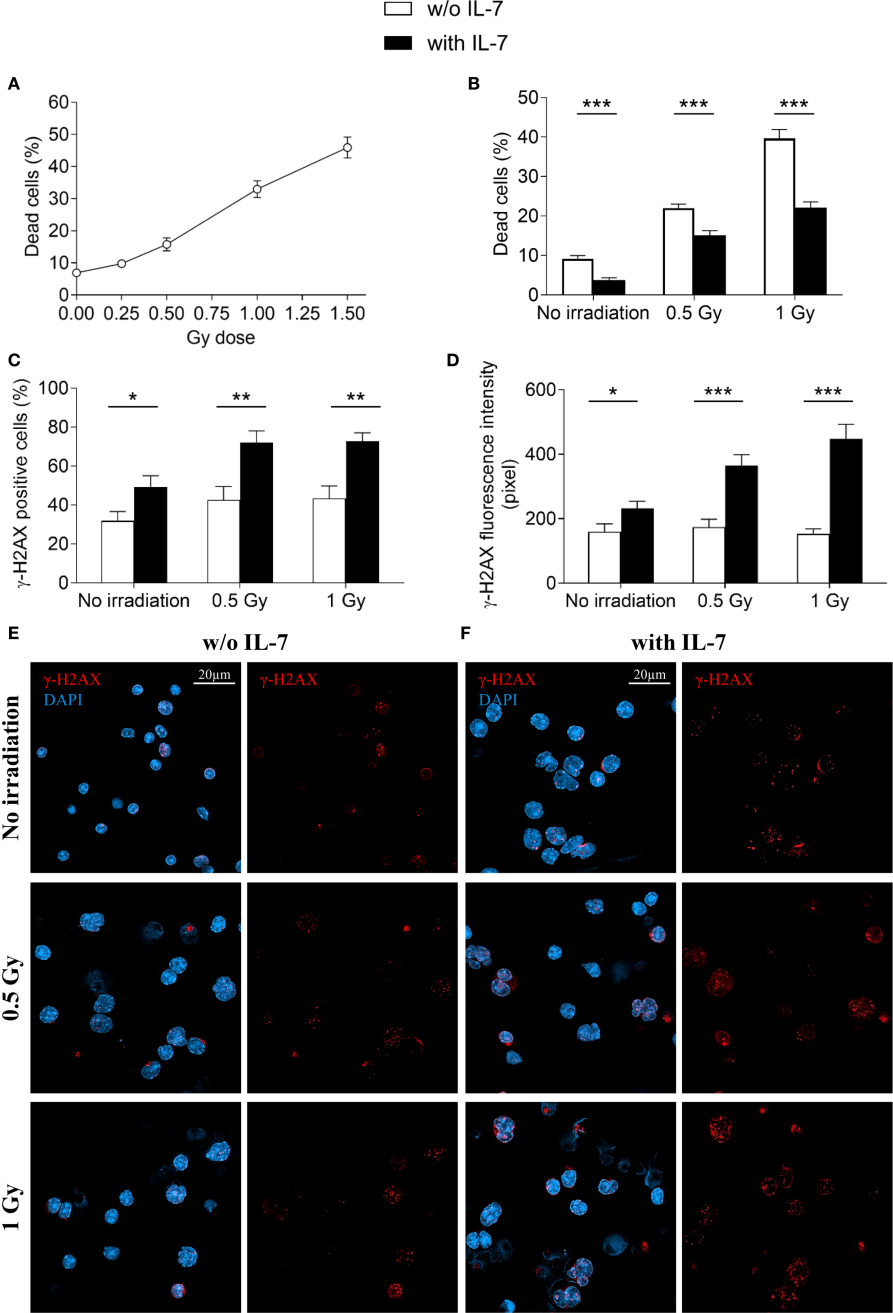
Effects of IL-7 treatment on irradiated pro-B cells. Pro-B cells were irradiated and cultured without or with IL-7 (50 ng/ml); cell survival and γ-H2AX foci were analyzed at 24 hours as described in Material and Methods. **(A)** Percentage of dead cells in irradiated pro-B cells (0.25, 0.5, 1 and 1.5 Gy). **(B)** Percentage of dead cells in untreated (white bars) and IL-7 treated (black bars) irradiated pro-B cells (0.5 and 1 Gy). **(C)** Percentage of γ-H2AX positive cells. **(D)** γ-H2AX fluorescence intensity. **(E)** Representative images of γ-H2AX immunofluorescence staining in untreated irradiated pro-B cells (0.5 and 1 Gy). **(F)** Representative images of γ-H2AX immunofluorescence staining in IL-7 treated irradiated pro-B cells (0.5 and 1 Gy). γ-H2AX foci in red, nuclei in blue. Right panel: γ-H2AX staining alone, left panel: merge. Results represent mean of 3 experiments ± SEM. The number of cells analyzed in the three independent experiments is: with IL-7 n= 164, 129 and 111 for non-irradiated samples, 0.5 Gy and 1 Gy, respectively; without IL-7 n= 115, 113 and 136 for non-irradiated samples, 0.5 Gy and 1 Gy, respectively. Unpaired *t* test, ***P < 0.001 **P < 0.01 and *P < 0.05. Scale bar: 20 µm.

We further investigated the effect of IL-7 on the formation of γ-H2AX foci and cell survival upon irradiation. Without IL-7, at 24 hours, irradiation of pro-B cells (0.25, 0.5, 1 and 1.5 Gy) caused dose-dependent cell death, with 46% reduction of cell survival at the highest dose (**Figure 8A**). We then assessed the effects of 0.5 and 1 Gy doses alone and in combination with IL-7. **Figure 8B** shows that IL-7 reduced cell death from 22% to 15% at 0.5 Gy dose and from 40% to 22% at 1 Gy dose (** *P* < 0.001 and *** *P* < 0.0001; respectively). However, IL-7 increased the percentage of radiation-induced γ-H2AX positive cells (* *P* < 0.01; **Figure 8C**), and γ-H2AX fluorescence intensity (* *P* < 0.001; **Figure 8D**). The number of γ-H2AX foci per nucleus and the nuclear area were also increased (not shown).

Representative images show γ-H2AX staining of untreated (**Figure 8E**) and IL-7-treated pro-B cells (**Figure 8F**) at the indicated Gy doses. These results indicate that while IL-7 enhanced the formation of radiation-induced DSBs, it also supported cell survival.

## 4 Discussion

In this study, we demonstrated that, in pro-B cells, IL-7 promoted the formation of DNA double-strand breaks (DSBs) and induced the expression of proteins involved in homologous recombination.

The results of a gene expression analysis suggested that IL-7 increased the transcription of genes involved in DNA repair. We focused on 5 proteins: RAD51, PLK1, BARD1, BRIP1 and MYC. RAD51, in conjunction with BRCA2, performs the DNA template homology search, which is fundamental in HR (25). The serine threonine kinase PLK1 phosphorylates RAD51, facilitating its recruitment at DSBs (34). The BARD1/BRCA1 protein complex recruits and stabilizes RAD51; in addition, it functions as an E3 ubiquitin ligase, and regulates the activity of many substrates, including histones (35). The helicase BRIP1, complexed with BRCA1, unwinds DNA at sites of damage (36). Our data are consistent with previous reports showing that MYC expression is induced by IL-7 in pro-B cells (2). MYC associates with the *Brca2* gene promoter in B cell lines, and with the *Exo1*, *Rad9*, *Rad50* and *Rad54L* promoters in Burkitt lymphoma cells (37, 38). Importantly, *Myc* silencing impairs the repair of radiation-induced DSBs (39). We observed that IL-7 increased transcription and protein expression in parallel. The upregulation of RAD51, PLK1 and MYC proteins preceded that of BARD1 and BRIP1, following a time course consistent with the sequence of HR steps (25). Our data suggest that proliferating pro-B cells undergoing V_H_(D)J_H_ recombination utilize HR, a pathway that maintains genomic stability. The positive effect of IL-7 on DNA repair gene expression may not be limited to proliferating cells, as preliminary data indicate increased mRNA levels of these genes in non-proliferating lymphoid cells. RAG-dependent DSBs are thought to be repaired by NHEJ (40). However, the studies cited in this comprehensive review concerned small pre-B cells recombining κ and λ light chain loci during the G1 phase, and pro-B cells undergoing the alternative NHEJ pathway, that are blocked in G0/G1 phase (40). Cell cycle phases, resection, template availability, transcriptional status, and the extent and type of DNA injury markedly influence the choice of the repair pathway (25, 41, 42). IL-7 is a powerful inducer of pro-B cell proliferation, and DSBs occurring in the G2-M phases are usually repaired by HR (2, 4, 25). In addition, in mammalian cells, HR and NHEJ can coexist and complement each other (43, 44). Although further experiments are needed to provide direct evidence that RAG-induced DSBs are repaired by HR, previous studies have shown that RAG-mediated nicks can efficiently initiate homologous recombination (45). We observed that IL-7 increased the percentage of γ-H2AX positive pro-B cells, the number of foci per nucleus and γ-H2AX fluorescence intensity in a time-dependent manner. All parameters declined by 192 hours, suggesting that DSB repair occurred within this timeframe. We evaluated the effect of IL-7 in RAG-deficient mice. In the three mice strains, the presence of distinct cells populations reflects the heterogeneous expression of the γ-H2AX signal, which decreases as cells progress to more mature stages. IL-7 did not promote DSB formation in RAG1-deficient pro-B cells, indicating the requirement of the RAG1 subunit-dependent cleavage. In RAG2-deficient pro-B cells the formation of DSBs was altered. A dysregulated cleavage, likely performed by truncated RAG2 proteins, might be due to an incomplete knock-out of the gene. However, this cleavage was insufficient for effective recombination, as demonstrated by the absence of mature peripheral lymphocytes (not shown). We cannot exclude additional effects of IL-7, for instance a potential effect on H2AX histone phosphorylation, as suggested by the observed increase in γ-H2AX fluorescence, or on chromatin accessibility.

IL-7, like other common γ-chain cytokines, increases glucose metabolism and cell size in T cells (2). We observed an increase in cell size in IL-7 treated pro-B cells. This effect may be partly mediated by MYC, which promotes the transcription of many genes involved in glucose metabolism (46).

B cell lineage specification gradually progresses through differentiation stages characterized by the regulated expression of specific molecules (31, 32). CD43 surface expression inversely correlates with the degree of B lymphoid and erythroid cell differentiation (31, 32, 47, 48). Common lymphoid progenitors, pre-pro-B cells and pro-B cells express CD43 at high levels. Pro-B cells and large pre-B cells progressively reduce CD43 expression, thus, a fraction of large pre-B cells, small pre-B cells, immature B cells and mature B cells are CD43 negative (31, 32, 47). CD43 is a heavily glycosylated protein and has been hypothesized to function as either an adhesive or an antiadhesive molecule, depending on its glycosylation status (49). CD43 adhesion properties in B cell progenitors are unknown. Only one study has reported reduced tissue engraftment by a CD43-deficient leukemic B cell line (50). We observed that IL-7 upregulated CD43 expression. It remains to be investigated whether these changes modify the retention and/or the release of progenitor cells from the bone marrow niche. Thus, further studies are needed to clarify the role of CD43 in this context.

The effect of IL-7 on γ-H2AX expression was observed in larger, CD43-intensely positive, immature pro-B cells. This result is consistent with previous studies showing that IL-7 is an important regulator of *Ig_H_* VDJ recombination in the pro-B cell stage (4).

It has been shown that IL-7 antagonized radiation-induced cell death in murine intestinal epithelial cell progenitors, an effect attributed to the increased expression of anti-apoptotic proteins (51). In agreement with these results, we observed that pro-B cells were sensitive to low-dose irradiation and showed increased γ-H2AX foci formation and cell death in a dose-dependent manner. To the best of our knowledge, this is the first demonstration that the addition of IL-7 can increase the formation of γ-H2AX foci and simultaneously antagonize cell death. The converging effect of IL-7 on survival and DNA repair may protect progenitor cells from DSB-induced damage.

As previously mentioned, the PARP family enzyme PARP1 plays a major role in promoting DNA repair (27, 28). The upregulation of DNA repair is exploited by neoplastic cells for chemo- and radio-resistance (27, 28). In BRCA1/2-mutated breast and ovarian cancers, PARP1 inhibitors are currently used in combination with chemotherapy to target DNA repair mechanisms, as homologous recombination (HR)-deficient cells cannot survive additional impairments in DNA repair (27, 28). IL-7 promotes the proliferation and survival of TALL and IL-7R+ BALL cells (2). Moreover, 10% of TALL, and occasionally other hematological malignancies harbor gain-of function mutations in IL-7Rα chain (2). IL-7-driven regulation of HR may thus preserve genomic stability during B-cell development and have potential clinical relevance (52, 53). Our data suggest that targeting the IL-7 and PARP-mediated effects on DNA repair could improve therapeutic strategies for acute leukemia. The evidence that the survival of TALL cells with HR defects is impaired by PARP inhibitors may support this hypothesis (54).

## Data Availability

All relevant data is contained within the article: the original contributions presented in the study are included in the article/**Supplementary Material**. Further inquiries can be directed to the corresponding author.
