# Supplementary material for: IL-7 promotes the formation of DNA double strand breaks and DNA repair in murine pro-B cells
Source: Front Immunol. 2025 Oct 6;16:1633892. doi: 10.3389/fimmu.2025.1633892 (PMC12535990; doi:10.3389/fimmu.2025.1633892)
Supplement: Supplementary file 1 [file DataSheet1.docx]

Supplementary Material

# Supplementary Tables

# Supplementary Table 1. Primer sequences for quantitative Real-Time PCR analysis of mRNA expression in murine pro-B cells.
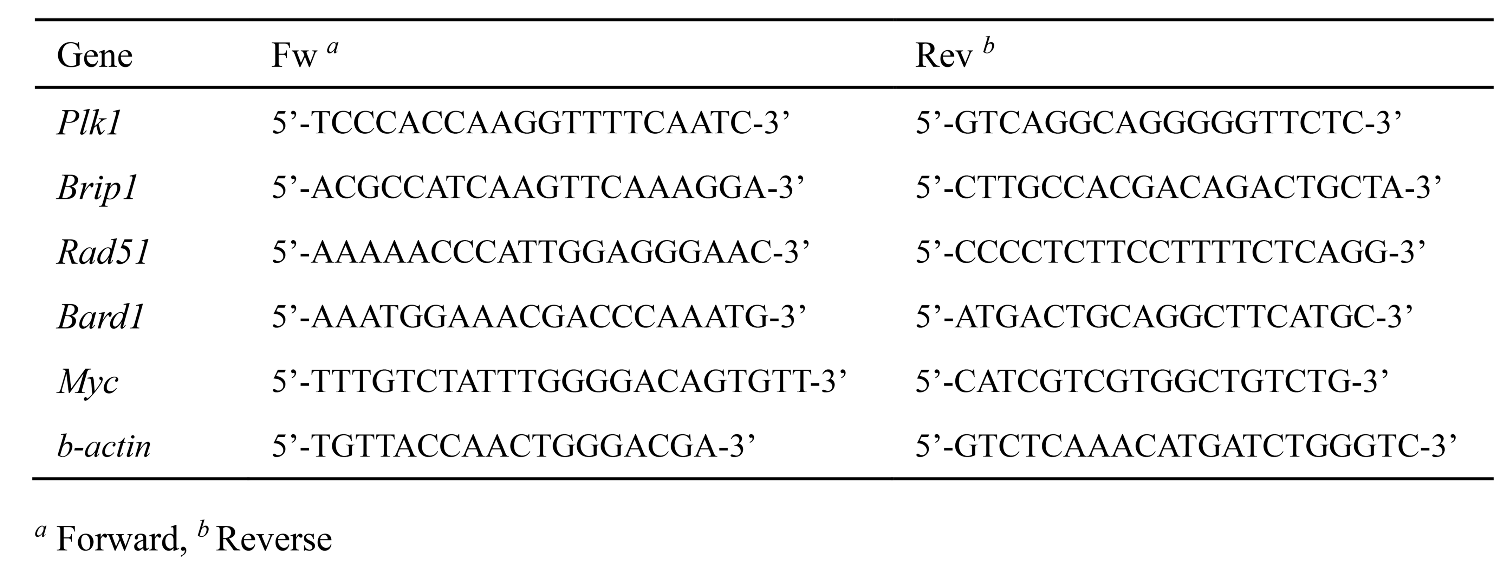


# Supplementary Table 2. List of 1159 IL-7-regulated genes (725 upregulated and 434 downregulated genes) in murine pro-B cells.

# Supplementary Table 3. Evaluation of γ-H2AX foci in pro-B cells after 96 and 144 hours of IL-7 treatment.


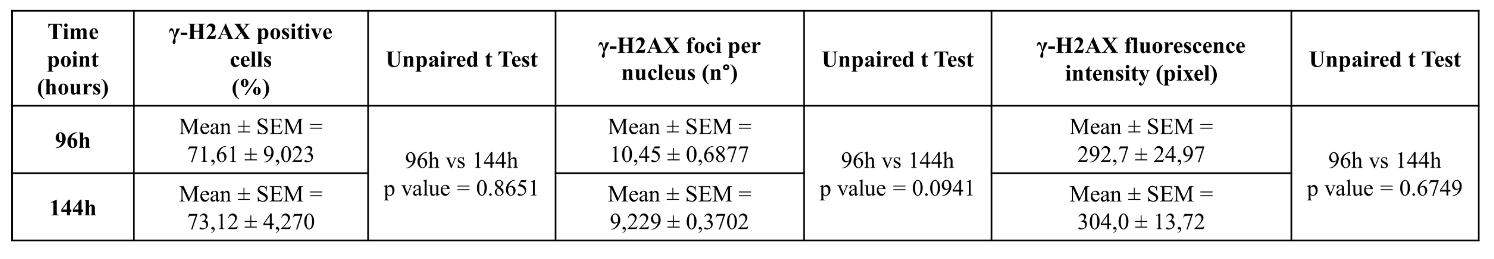


# Supplementary Table 4. CD43 expression of pro-B cells untreated or IL-7-treated for 24, 48 and 72 hours in three independent experiments.


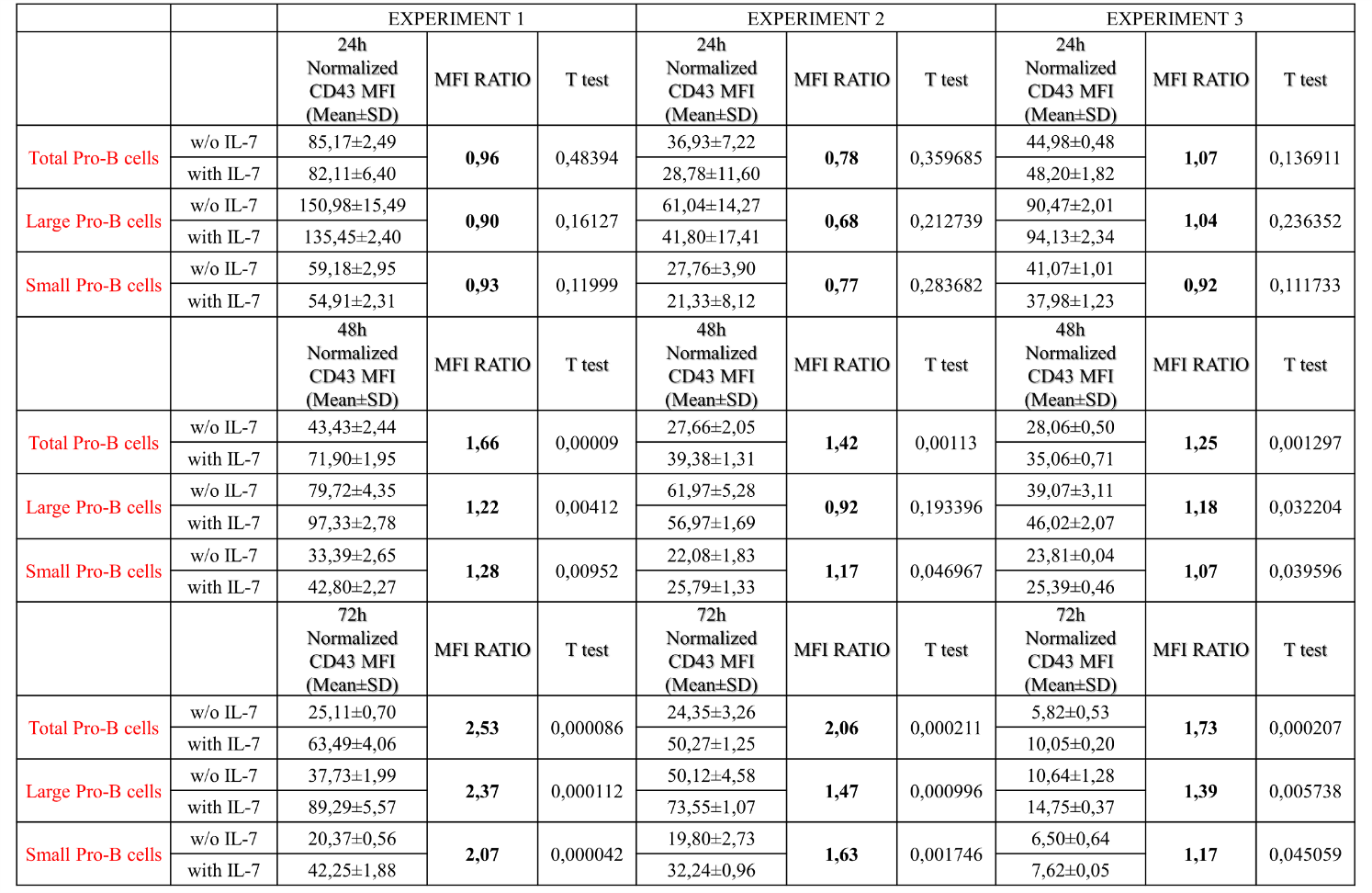


## Supplementary Figures

##
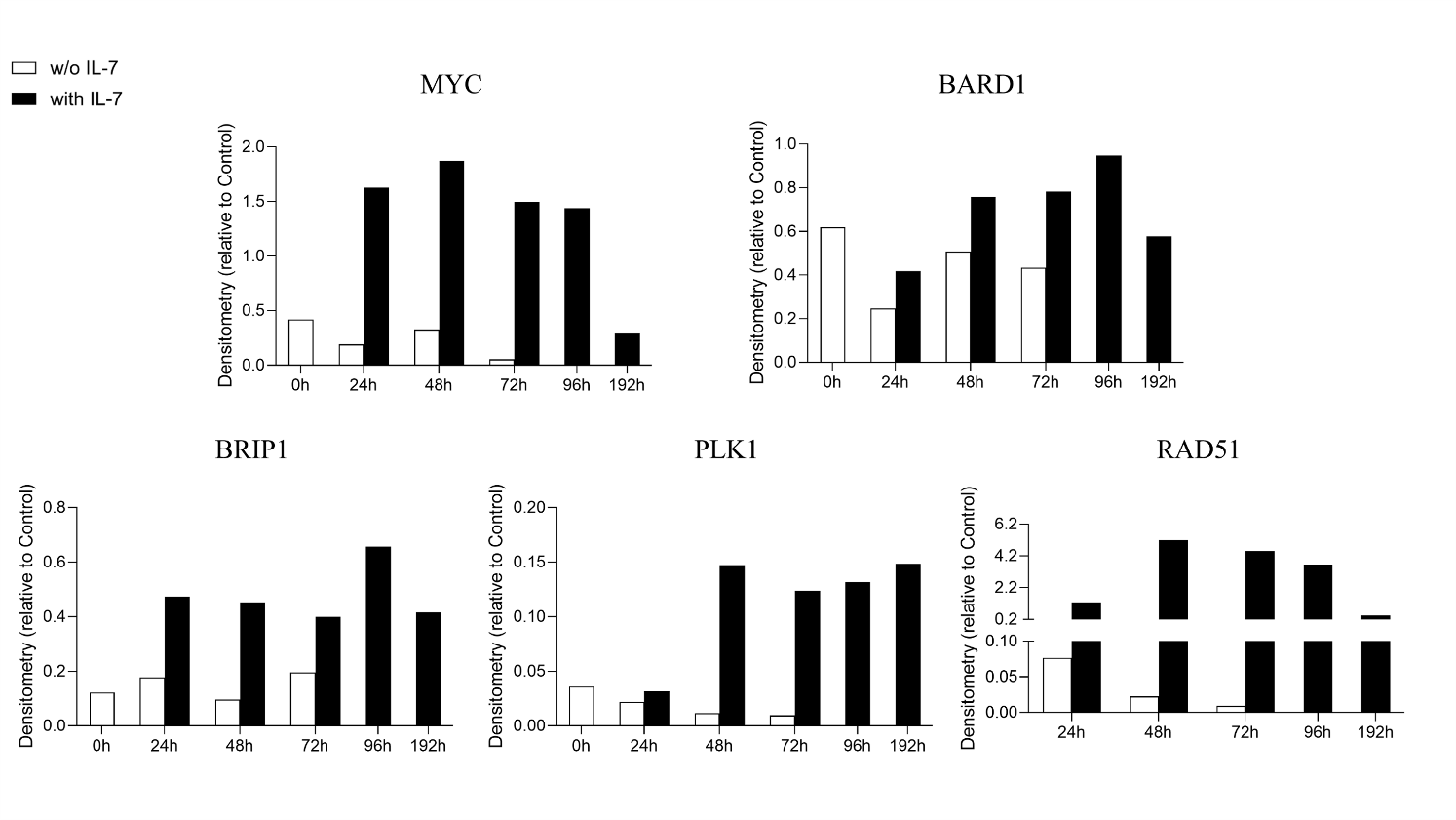


**Supplementary Figure 1.** Western blot densitometric analyses from Fig. 1E-F: each band intensity was analyzed by ImageJ and normalized to that of the loading control (β-actin).

**
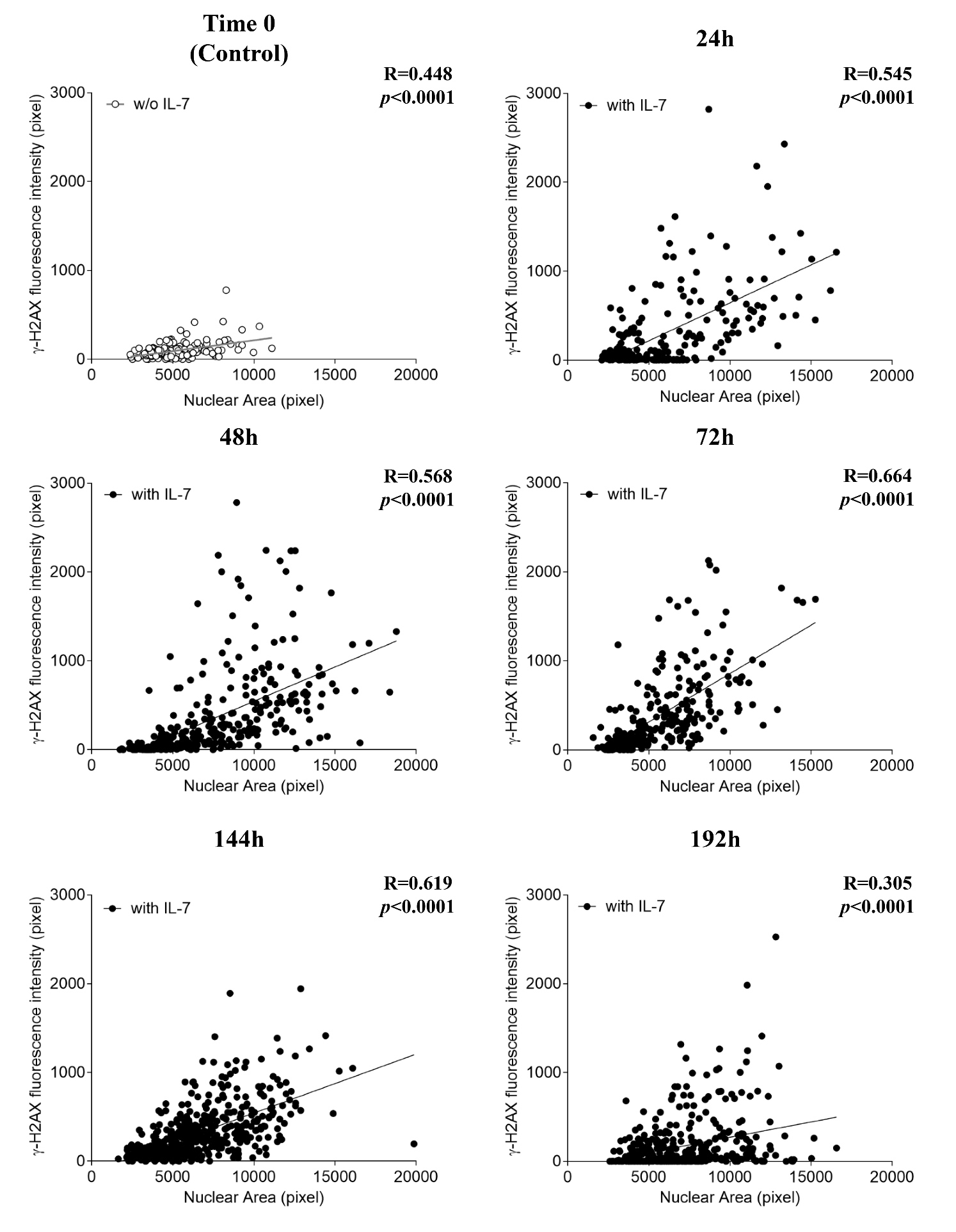
**

**Supplementary Figure 2.** IL-7 markedly increased γ-H2AX mean fluorescence intensity in large nuclei. Pro-B cells, except freshly isolated control cells (Time 0), were cultured with IL-7 (50 ng/ml) for the indicated times. Linear regression (Pearson’ correlation test) was used to correlate γ-H2AX mean fluorescence intensity and nuclear area (each dot represents a nucleus). R and P value are reported in each panel.

**
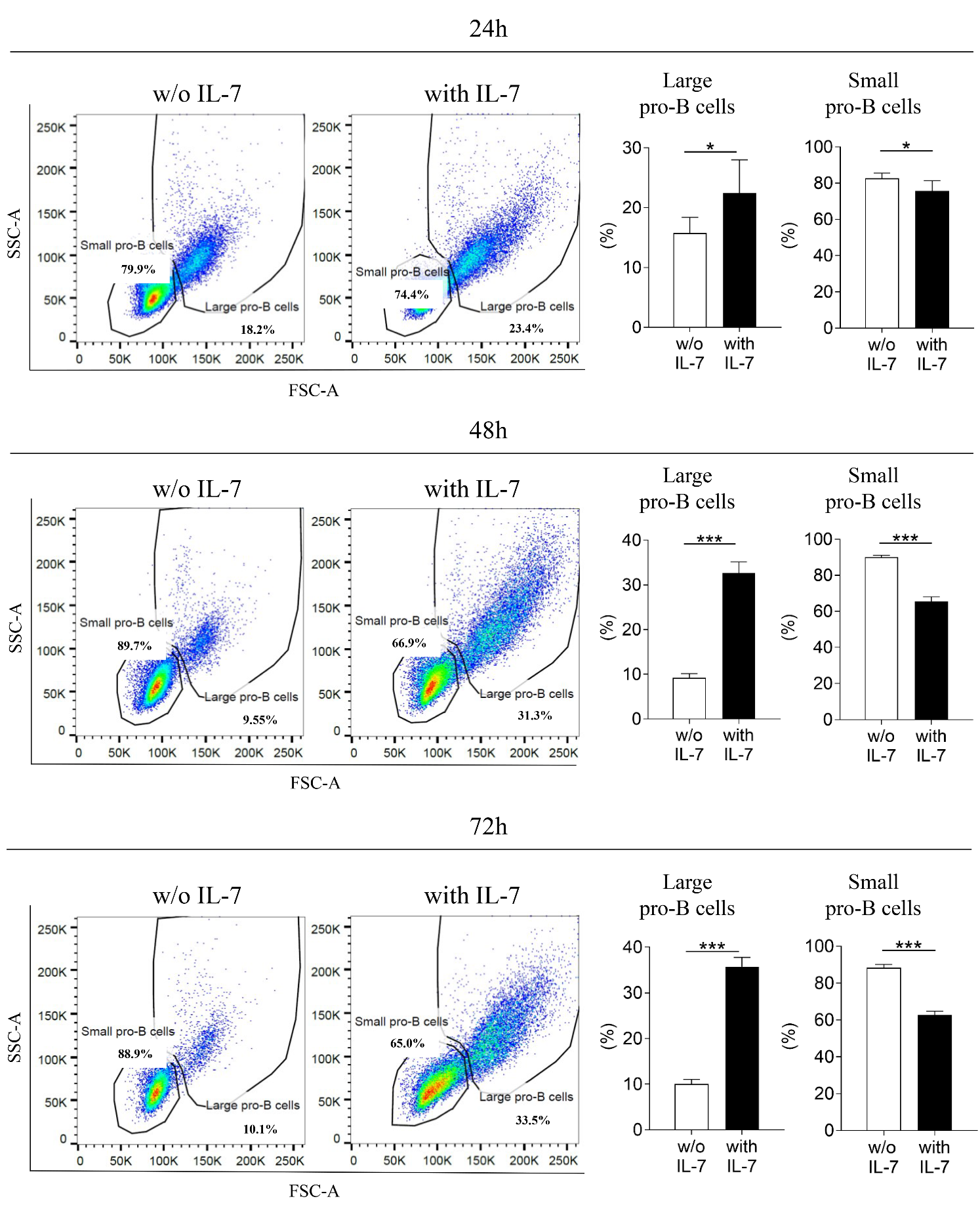
**

**Supplementary Figure 3.** IL-7 markedly increased the percentage of large pro-B cells in a time dependent manner. Pro-B cells were cultured without IL-7 or with IL-7 (50 ng/ml) for the indicated times. Forward and side scatter were evaluated as described in Materials and Methods. Scatterplots showed 1 of 3 experiments, with bar plots representing mean ± SD of 3 experiments. IL-7 increased the percentage of large cells from 15.77 ± 2.62% to 22.45 ± 5.56% at 24 hours (* P < 0.05, upper panel), from 9.23 ± 0.92% to 32.65 ± 2.48% at 48 hours (*** P < 0.0001, middle panel) and from 10.01 ± 1.05% to 35.72 ± 2.07% at 72 hours (*** P < 0.0001, lower panel). After IL-7 treatment, small cells percentage significantly decreased from 82.65 ± 2.91% to 75.67 ± 5.66% at 24 hours (* P < 0.05, upper panel), from 90.07 ± 0.96% to 65.48 ± 2.57% at 48 hours (** P < 0.0001, middle panel) and from 88.38 ± 1.82% to 62.62 ± 2.19% at 72 hours (*** P < 0.0001, lower panel). Unpaired t test.


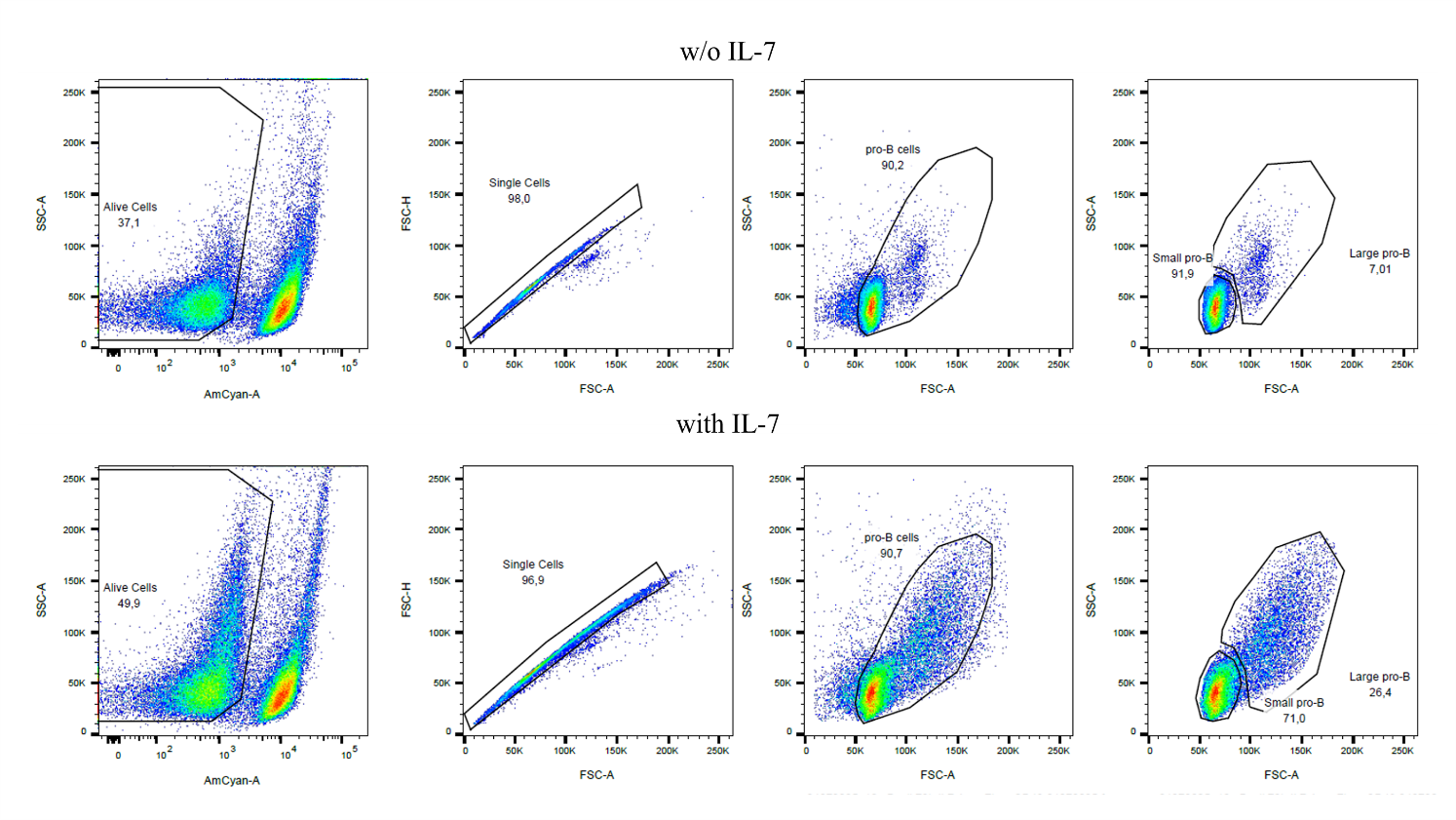


**Supplementary Figure 4.** Pro-B cells were treated without or with IL-7 for 72 hours and stained with the LIVE/DEAD™ Fixable Aqua Dead Cell Stain Kit. Cytograms show the gates used to separate live/dead cells and large/small cells.
